# A Multicountry Ecological Study of Cancer Incidence Rates in 2008 with Respect to Various Risk-Modifying Factors

**DOI:** 10.3390/nu6010163

**Published:** 2013-12-27

**Authors:** William B. Grant

**Affiliations:** Sunlight, Nutrition, and Health Research Center, P.O. Box 641603, San Francisco, CA 94164-1603, USA; E-Mail: wbgrant@infionline.net; Tel.: +1-415-409-1980

**Keywords:** cancer incidence rates, diet, ecological, latitude, smoking, ultraviolet-B, vitamin D

## Abstract

Observational and ecological studies are generally used to determine the presence of effect of cancer risk-modifying factors. Researchers generally agree that environmental factors such as smoking, alcohol consumption, poor diet, lack of physical activity, and low serum 25-hdyroxyvitamin D levels are important cancer risk factors. This ecological study used age-adjusted incidence rates for 21 cancers for 157 countries (87 with high-quality data) in 2008 with respect to dietary supply and other factors, including per capita gross domestic product, life expectancy, lung cancer incidence rate (an index for smoking), and latitude (an index for solar ultraviolet-B doses). The factors found to correlate strongly with multiple types of cancer were lung cancer (direct correlation with 12 types of cancer), energy derived from animal products (direct correlation with 12 types of cancer, inverse with two), latitude (direct correlation with six types, inverse correlation with three), and per capita gross national product (five types). Life expectancy and sweeteners directly correlated with three cancers, animal fat with two, and alcohol with one. Consumption of animal products correlated with cancer incidence with a lag time of 15–25 years. Types of cancer which correlated strongly with animal product consumption, tended to correlate weakly with latitude; this occurred for 11 cancers for the entire set of countries. Regression results were somewhat different for the 87 high-quality country data set and the 157-country set. Single-country ecological studies have inversely correlated nearly all of these cancers with solar ultraviolet-B doses. These results can provide guidance for prevention of cancer.

## 1. Introduction

Cancer is one of the leading causes of disease and death worldwide. In 2008, an estimated 12.66 million new cases of cancer occurred, excluding nonmelanoma skin cancer, and 7.56 million cancer deaths (approximately 13% of all deaths) [1]. The most frequent types were lung, prostate, colorectal, stomach, and liver cancer for men, and breast, colorectal, cervix uteri, lung, and stomach cancer for women. However, in the United States in 2011, cancer deaths accounted for 23% of all deaths [2,3].

Although much of the war on cancer emphasizes early detection and treatment, the burden of cancer will remain high unless the primary risk factors for cancer are understood and addressed. A general understanding exists of many of the cancer risk—modifying factors; for example, according to the World Health Organization (WHO) [4].

About 30% of cancer deaths are due to the five leading behavioral and dietary risks—high body mass index, low fruit and vegetable intake, lack of physical activity, tobacco use, and alcohol use.

- Tobacco use is the most important risk factor for cancer, causing 22% of global cancer deaths and 71% of global lung cancer deaths.
- Cancer-causing viral infections such as hepatitis B and C viruses and human papillomavirus are responsible for up to 20% of cancer deaths in low- and middle-income countries.

The American Institute for Cancer Research/World Cancer Research Fund [5] recommends limiting intake of red meat and avoiding processed meat, eating mostly foods of plant origin, and limiting consumption of energy-dense foods, which includes sugary drinks.

Several epidemiological approaches are used to investigate cancer risk-modifying factors. These approaches include case-control studies, nested case-control studies derived from cohort studies, and ecological studies. Multicountry ecological studies have been used to identify and quantify risk factors for cancer. In 1975, examined dietary links to cancer by using incidence data for 27 cancers from 23 countries and mortality rates for 14 cancers from 32 countries [6]. They found that meat consumption was an important risk factor for colon cancer and that fat consumption was an important risk factor for breast and endometrial cancer. Per capita gross domestic product (GDP) also highly correlated with these cancers.

Both single- and multicountry ecological studies offer evidence that solar ultraviolet-B (UVB) irradiance, through production of vitamin D, reduces cancer risk [7,8,9,10]. The UVB–vitamin D–cancer hypothesis has received reasonable support in other types of studies, including case-control and nested case-control studies, and the mechanisms whereby vitamin D reduces risk of cancer are well known [11,12]. The UVB–vitamin D–cancer hypothesis is thought to generally satisfy the criteria for causality in a biological system outlined by Hill [13] for several cancers [14,15].

Using the ecological study approach, this study presents results with cancer incidence data for 87 countries with high-quality mortality rate data and 157 countries total with respect to dietary supply and other factors, including per capita GDP, life expectancy, smoking, and latitude (an index for solar UVB doses). I then compare the results to published results from observational studies.

## 2. Data and Methods

The hypothesis tested in this study is that various cancer risk-modifying factors affect cancer incidence rates at the national population level and the relative contributions to cancer risk can be determined. The risk-modifying factors considered in this study include alcohol consumption, animal fat consumption, animal product consumption, cereals consumption, gross domestic product, life expectancy, solar UVB and vitamin D production, and sweeteners consumption. Other components of diet, such as eggs, fish, meat, and milk, were considered, but since they are highly correlated with total animal product supply, it was not though that any one of these factors would alone explain the finding for total animal product supply. Other cancer risk-modifying factors were not considered; generally due to the fact that it would be difficult to obtain or develop and index that could be used for all countries. Some of the factors omitted are discussed later.

Cancer incidence data for 2008 came from GLOBOCAN [1]. I chose countries used in this study according to two criteria. First, the country must have dietary supply data from the Food and Agriculture Organization published in printed form for 1992–1994 [16]. The reason for this requirement is that diet may affect the risk of cancer 10–30 years before cancer develops [17,18]. Second, the country must have more than 100,000 inhabitants in 1992–1994 since developing robust cancer statistics for countries with fewer inhabitants is difficult. A total of 157 countries met both criteria. Of those, 87 countries have level 1 or 2 quality data (hereafter, high-quality data), on the basis of classifications by WHO for deaths in 2001–2002 [19,20]. Appendix lists countries used in this study.

Dietary supply data came from the Food and Agriculture Organization, both from the book published in 1996 for 3-year averaged data from 1979–1981 to 1992–1994 [16] and from their website for single-year averaged data for 1995 and 2000 [21]. I obtained values for alcohol, animal fat, animal product energy, cereals, energy (total), fat, fish, and meat. I used data for various years since a 10- to 30-year lag generally exists between national dietary changes and cancer incidence rate [17,18]. For example, animal product energy supply increased in the 1990s for some countries but decreased in other countries; varying the year of the data serves to help estimate the lag between dietary change and cancer incidence.

I used lung cancer incidence rates as the index of health effects of smoking. Ecological studies of cancer in Nordic countries have used lung cancer incidence rates [22], and ecological studies in the United States [8] and Spain [23] have used lung cancer mortality rates. Lung cancer incidence or mortality rates are ideal since they integrate the effects of smoking over many years [24]. Also, cigarette consumption data are not available for all countries. A possible downside is that diet and other factors, such as solar UVB doses/vitamin D [25], can also affect risk of lung cancer. However, as results for various cancers will show, this does not present a problem.

Nonetheless, I obtained data for cigarette consumption per capita for 1990 from WHO, generally of the form used on its data sheet for Poland [26], with the country name changed for different countries. Some URLs were of a slightly different form and found through additional Internet searches. I chose the year 1990 since lung cancer takes many years to develop and I judged the data for 2000 to be too recent. I could have used data for 1980 although fewer countries have data for 1980, but the data for 1990 serve the intended purpose to show that lung cancer is strongly linked to cigarette consumption. I obtained data for 71 countries, including 54 countries with high-quality cancer incidence data and life expectancy greater than 60 years for males in 1990. I apportioned the cigarette consumption data to males and females in proportion to the male–female balance of lung cancer incidence rates in each country in 2008.

The index used for socioeconomic status is GDP per capita for 1999 from the International Monetary Fund [27]. Data were available for 143 countries. However, the analyses omitted data for one country, Brunei, since the high per capita GDPs for this country were acquired recently and probably have not been shared with many in the country’s population, such as guest workers. I considered two other sets of data: International Monetary Fund data for 1989 [27] and CIA values for 2008 [28]. Among concerns regarding the 1989 data is that many countries were not included, in part since they were not independent countries then, and that 1989 was 19 years before the cancer incidence data. One problem with the 2008 data is that the data are for the same year as for cancer incidence rates. However, GDP per capita for the year of cancer incidence could reflect cancer screening rates for breast, colorectal, and prostate cancer.

The analyses also used latitude of the approximate center of population of each country. This index is considered an index of annual solar UVB doses.

I obtained life expectancy data for males in 1990 for 151 countries from World Health Statistics [19]. For some cancers, cancer rates correlated with life expectancy for countries with life expectancy greater than 60 years.

I analyzed the countries in three different sets: the entire set (157 countries); that for countries with life expectancy of 60 years or greater for males in 1990 (100 countries); and countries with high-quality data in 2001 and 2002 for causes of death according to [15,19], along with life expectancy greater than 60 years for males in 1990 (87 countries). I estimated cancer incidence rates for the remaining 70 countries from data for countries with high-quality data [20]. Thus, rates are not entirely independent of rates in countries with high-quality data; however, values for risk-modifying values should be independent. When the incidence rate was zero, I omitted the value. KaleidaGraph [29] created the graphs.

I ran regression analyses by using IBM SPSS Statistics 20 (Armonk, New York, NY, USA). The analyses included various combinations of risk-modifying factors for each type of cancer and for both sexes. I eliminated those factors with large *p* values and reran the regression. For factors that had two or more sets of data for various years, such as animal products, cereals, and sweeteners, I used pairs of data to see which had the stronger correlation. This was a useful step since not all countries had values for each year. When multiple linear regression analysis found significantly correlated factors, I ran each factor in a linear regression analysis to see whether each was independently significantly correlated with cancer incidence rates and with the same direction (direct or inverse) since the factors are highly correlated and can interfere with each other. The final analysis omitted those not independently correlated.

Table 1 lists cross-correlation coefficients for various risk-modifying factors for the 87 countries with high-quality data. Most factors are highly correlated with each other, except sweeteners. Latitude is highly correlated with energy from animal products (0.74 < *r* < 0.79). Cereals are inversely correlated with all other factors.

## 3. Results

Table 2 gives regression results for the set of 87 high-quality countries. Significant results emerged for 21 cancers as well as all and all less lung cancer for countries with high-quality data. The regression found no significant results for esophageal, gallbladder, and stomach cancer.

Table 3 gives regression results for all 157 countries. The regression results for the 100 countries with male life expectancy greater than 60 years in 1990 were similar to those for the other two sets of countries (data not shown).

Table 4 gives the numbers of cancers significantly correlated with each risk-modifying factor for the two sets of countries. Four factors have significant correlations with a reasonable number of cancer types: lung cancer, energy derived from animal products, latitude, and GDP. Alcohol supply is associated only with colorectal cancer.

The results for animal product energy show that data for 1990 have the most high-significance correlations with cancer incidence, indicating about a 15- to 20-year lag between diet and cancer incidence. Prostate cancer has the longest lag, 28 years.

Life expectancy is significantly associated with several cancers: brain and colorectal cancer, leukemia, and multiple myeloma.

Latitude is directly correlated with several cancers: bladder, brain, kidney, lung (females), melanoma, and Hodgkin’s lymphoma, and inversely correlated with cervical, lip, and thyroid cancer. Latitude is generally not found significantly correlated with cancer incidence rates if animal product energy is; this is the case for 11 cancers. Both animal product energy and latitude were significantly correlated for only two cancers: brain and kidney cancer. Evidently when energy from animal products is significantly correlated with cancer incidence rates, it overwhelms the effect of latitude.

Lung cancer was directly correlated with cigarette consumption per capita for both sexes as well as animal fat in 1993 and latitude.

The regression results for all cancer less lung cancer for both males and females for high-quality countries found a significant correlation between energy derived from animal products and lung cancer. Alcohol and latitude were also directly correlated with all cancer less lung cancer for females, whereas cereals were inversely correlated.

## 4. Discussion

The lag of 15–20 years between energy derived from animal products is supported from studies of cancer trends in countries undergoing nutrition transitions: 15–27 years for colon cancer mortality rates in Japan [30], 20–31 years for breast cancer mortality rates in Japan [18], and 10 years for mortality rates for several cancers in several Southeast Asian countries [19]. Studies reporting early-life effects on risk of prostate cancer [31,32] support the finding of 28 years for prostate cancer.

Other evidence supporting the role of energy derived from animal products as important risk factors includes the finding that Seventh Day Adventists, who generally are lacto-ovo vegetarians who do not smoke or drink alcohol excessively, have lower cancer rates than do others living in the same region [33,34,35].

**Table 1:** Cross-correlations of factors used in this study for the 87 high-quality countries *.

Factor	Animal Energy, 1980	Animal Energy, 1985 (*r*, *r*^2^, *p*)	Animal Energy, 1990 (*r*, *r*^2^, *p*)	Animal Energy, 1993 (*r*, *r*^2^, *p*)	Animal Energy, 2000 (*r*, *r*^2^, *p*)	Animal Fat, 2000 (*r*, *r*^2^, *p*)	Cereals 1993 (*r*, *r*^2^, *p*)
Alcohol, 1993	0.62, 0.37	0.66, 0.43	0.71, 0.49	0.63, 0.38	0.65, 0.41	0.67, 0.44	−0.51, 0.24
Animal en, 1980		0.99, 0.98	0.96, 0.92	0.94, 0.87	0.94, 0.87	0.92, 0.85	−0.59, 0.34
Animal en, 1985			0.98, 0.96	0.96, 0.92	0.94, 0.88	0.95, 0.90	−0.61, 0.36
Animal en, 1990				0.99, 0.97	0.95, 0.89	0.95, 0.90	−0.61, 0.36
Animal en, 1993					0.95, 0.91	0.95, 0.90	−0.58, 0.33
Animal en 2000						0.99, 0.98	−0.60, 0.36
Animal fat, 2000							−0.60, 0.35
**Factor**	**Fat, 2000 (*r*, *r*^2^, *p*)**	**GDP 1999 (*r*, *r*^2^, *p*)**	**Latitude (*r*, *r*^2^, *p*)**	**Life Expectancy (*r*, *r*^2^, *p*)**	**Lung Cancer, M (*r*, *r*^2^, *p*)**	**Lung Cancer F (*r*, *r*^2^, *p*)**	**Sweeteners 1990 (*r*, *r*^2^, *p*)**
Alcohol, 1993	0.63, 0.39	0.57, 0.31	0.51, 0.25	0.47, 0.21	0.53, 0.27	0.45, 0.19	0.13, 0.001, 0.30
Animal en, 1980	0.81, 0.64	0.74, 0.54	0.79, 0.63	0.63, 0.38	0.61, 0.36	0.73, 0.53	0.29, 0.07, 0.02
Animal en, 1985	0.84, 0.69	0.77, 0.59	0.79, 0.61	0.66, 0.43	0.63, 0.39	0.72, 0.51	0.28, 0.07, 0.02
Animal en, 1990	0.85, 0.72	0.76, 0.57	0.77, 0.60	0.67, 0.43	0.65, 0.42	070, 0.49	0.23, 0.04, 0.06
Animal en, 1993	0.85, 0.72	0.75, 0.55	0.67, 0.44	0.65, 0.42	0.51, 0.25	0.65, 0.41	0.17, 0.02, 0.16
Animal en 2000	0.89, 0.79	0.79, 0.62	0.66, 0.43	0.66, 0.43	0.51, 0.25	0.67, 0.45	0.17, 0.02, 0.16
Animal fat, 2000	0.89, 0.78	0.77, 0.59	0.66, 0.43	0.63, 0.39	0.51, 0.25	0.67, 0.45	0.17, 0.01, 0.18
Cereals, 1993	−0.53, 0.27	−0.58, 0.33	−0.14, 0.008, 0.19	−0.62, 0.38	−0.23, 0.04, 0.04	−0.50, 0.24	−0.35, 0.11, 0.003
Fat, 2000		0.83, 0.68	0.53, 0.27	0.73, 0.53	0.46, 0.20	0.65, 0.41	0.14, 0.004, 0.27
GDP, 1999			0.41, 0.16	0.77, 0.59	0.26, 0.06, 0.02	0.67, 0.44	0.37, 0.12, 0.003
Latitude				0.24, 0.05, 0.02	0.63, 0.39	0.47, 0.21	—
Life expectancy					0.30, 0.08, 0.005	0.55, 0.29	0.29, 0.07, 0.02
Lung cancer, M, 2008						0.49, 0.23	—
Lung cancer, F, 2008							0.32, 0.09, 0.007

—: no significant results; *: if *p* < 0.001, value omitted; F: female; M: male; Animal en, Animal energy. After obtaining results of the regression analyses, I searched the journal literature through PubMed.gov for papers that supported the findings for risk-modifying factors for each type of cancer. The search was not meant to be comprehensive or to provide enough information to conclude whether the support could be considered definitive.

**Table 2:** Regression results for 87 high quality countries.

Cancer	Sex	Number of Countries	Lung Cancer	Animal Energy	Latitude	GDP 1999	Miscellaneous	Adjusted
			(β, *p*)	(year; β, *p*)	(β, *p*)	(β, *p*)	(β, *p*)	r^2^, *F*, *p*
All	M	82	0.40, *	2000; 0.44, *			Alc93: 0.23, 0.003	0.77, 93, *
		84	0.38, *	1993; 0.46, *			Alc93: 0.20, 0.997	0.77, 91, *
	F	87	0.49, *	1993; 0.33, *			Cer93: −0.20, 0.002	0.77, 99, *
		82	0.53, *	1993; 0.30, *			Alc93: 0.30, 0.004	0.77, 93, *
All Less Lung	M	62	0.23, 0.01	1980; 0.54, *		0.21, 0.04		0.74, 58, *
		87	0.28, *	1993; 0.52, *			Cer93: −0.21, 0.006	0.69, 63, *
	F	62	0.40, *	1990; 0.44, *		0.10, 0.38		0.72, 52, *
		82	0.43, *	1993; 0.34, *			Alc93: 0.22, 0.004	0.71, 67, *
		82	0.43, *		0.22, 0.004		Alc93: 0.20, 0.013Cer00: −0.25, 0.001	0.71, 51, *
Bladder	M	79	0.43, *		0.30, 0.003	0.23, 0.006		0.58, 37, *
	F	80	0.48, *		0.26, 0.003	0.17, 0.09		0.58, 37, *
Brain	M	79			0.58, *	0.26, 0.004		0.52, 44, *
		79		1993; 0.26, 0.004	0.58, *			0.51, 41, *
	F	85	0.33, 0.001		0.38, *			0.35, 24, *
Breast	F	80	0.23, 0.009	1993; 0.41, *		0.31, 0.002		0.72, 67, *
Cervix	F	80			−0.40, *	−0.33, 0.001		0.37, 24, *
Colorectal	M	77	0.40, *			0.31, *	Alc93: 0.39, *	0.75, 76, *
	F	67	0.37, *	1985; 0.17, 0.12			Alc93: 0.24, 0.003LE: 0.28, 0.001	0.77, 56, *
		82	0.33, *		0.25, 0.001		Alc93: 0.25, 0.001LE: 0.28, *	0.74, 58, *
Corpus uteri	F	68		1985; 0.67, *				0.44, 53, *
Hodgkin’s Lymphoma	M	82			0.25, 0.02		Fat00: 0.48, *	0.40, 28, *
	F	78			0.33, 0.005		Fat00: 0.29, 0.01	0.26, 15, *
Kidney	M	87	0.27, 0.004		0.50, *		Cer93: −0.25, 0.001	0.60, 44, *
		87	0.27, 0.003	1993; 0.32, 0.001	0.32, 0.003			0.60, 43, *
		87	0.40, *	1993; 0.47, *				0.56, 55, *
	F	85	0.30, 0.001		0.52, *		Cer93: −0.17, 0.04	0.60, 42, *
		85	0.30, 0.002	1993; 0.23, 0.04	0.39, *			0.60, 42, *
Laryngeal	M	85	0.86, *	2000; −0.33, *				0.55, 52, *
	F	78	0.48, 0.001	2000; −0.39, 0.008				0.12, 6, *
Leukemia	M	86	0.43, *				LE: 0.44, *	0.47, 39, *
		80	0.46, *			0.40, *		0.46, 35, *
	F	86	0.30, 0.006				LE: 0.36, 0.001	0.32, 21 *
Lip, oral	M	87	0.53, *					0.28, 34, *
	F	86	0.51, *		−0.31, 0.007			0.19, 11, *
Liver	M	67		1980; −0.27, 0.03				0.06, 5, 0.03
	F	67		1980; −0.51, *				0.25, 23, *
Lung	M	54	CigM: 0.65, *				AF93; 0.36, *	0.73, 72, *
		54	CigM: 0.63, *		0.35, *			0.72, 67, *
		54	CigM: 0.79, *					0.62, 89, *
		54	Cig: 0.80, *					0.62, 86, *
	F	54	CigF: 0.65,*				AF93; 0.32, *	0.77, 92, *
		54	CigF: 0.64, *		010, 0.35		AF93: 0.26, 0.018	0.77, 61, *
		54	CigF: 0.70, *		0.26, 0.003			0.75, 81, *
		54	CigF: 0.85, *					0.71, 131, *
					Lat × Lat			
Melanoma	M	53 **			0.41, *	0.52, *		0.72, 68, *
		54 ***			0.48, *	0.43, *		0.69, 59, *
		52 **			0.40, *	0.53, *		0.72, 67, *
		53 ***			0.45, *	0.47, *		0.70, 63, *
Multiple myeloma	M	64		85; 0.57, *			LE: 0.27, 0.02	0.59, 46, *
		58		85; 0.53, *		0.29, 0.04		0.59, 42, *
	F	77				0.44, *	LE: 0.37, 0.003	0.58, 54, *
		77				0.74, *		0.53, 88, *
Non-Hodgkin’s lymphoma	M	80				0.75, *		0.55, 97, *
	F	80				0.73, *		0.54, 91, *
Ovarian	F	68		85; 0.70, *				0.49, 65, *
Pancreatic	M	86	0.71, *	1993; 0.16, 0.04				0.64, 78, *
		86	0.80, *					0.63, 144, *
	F	86	0.40, 0.001	1993; 0.31, 0.008				0.39, 29, *
Prostate	M	68	−0.26, 0.014	80; 0.62, *			Cer93: −0.36, 0.001	0.59, 33, *
Testicular	M	59		85; 0.66, *		0.25, 0.023		0.74, 82, *
Thyroid	M	82		1993; 0.74, *	−0.29, 0.023			0.33, 20, *
	F	84		1993; 0.58, *	−0.25, 0.07			0.19, 10, *

*: *p* < 0.001; **: omits Australia, Luxembourg, New Zealand; ***: omits Australia, New Zealand; AF93: animal fat, 1993; Alc93: alcohol supply, 1993; Cer: cereals supply; Cer90: cereals supply, 1990; LE: life expectancy; F: female; M: male; CigF: cigarettes consumed by females; CigM: cigarettes consumed by males; Fat00: Fat, 2000.

**Table 3:** Regression results for the 157 countries.

Cancer	Sex	Number of Countries	Lung Cancer	Animal Energy	Latitude	GDP 1999	Miscellaneous	Miscellaneous	Adjusted
			(β, *p*)	(year; β, *p*)	(β, *p*)	(β, *p*)	(β, *p*)	(β, *p*)	*r*^2^, *F*, *p*
Bladder	M	140	0.40, *		0.36, *	0.19, 0.002			0.66, 91, *
	F	139	0.37, *		0.25, 0.001	0.22, 0.009			0.52, 50, *
Brain	M	126		1990; 0.26, 0.007	0.42, *			LE: 0.27, *	0.71, 101, *
		130	0.16, 0.036	1990; 0.37, *	0.38, *				0.68, 91, *
		145			0.59, *			LE: 0.35, *	0.67, 149, *
		130		1990; 0.44, *	0.44, *				0.67, 131, *
		126		1090; 0.58, *				LE: 0.27, 0.001	0.64, 111, *
	F	147	0.30, *		0.43, *			Sweet93: 0.18, 0.006	0.54, 59, *
		148	0.36, *		0.47, *				0.53, 83, *
Breast	F	142	0.22, *	1993; 0.49, *		0.26, 0.001			0.76, 147, *
		142	0.28, *		0.17, *	0.51, *			0.70, 111, *
Cervix	F	142			−0.40, *	−0.30, *			0.38, 44, *
Colorectal	M	122	0.38, *	1985; 0.21, 0.013		0.25, *		Alc93: 0.22, *	0.84, 160, *
		138	0.35, *		0.17, 0.007	0.26, *		Alc93: 0.31, *	0.80, 141, *
	F	131	0.36, *	1985; 0.31, *			LE: 0.14, 0.02	Alc93: 0.21, *	0.77, 112, *
		136	0.35, *	1985; 0.40, *				Alc93: 0.23, *	0.74, 127, *
Corpus uteri	F	137	0.38, *	1990; 0.37, *				Sweet90: 0.21, 0.001	0.68, 97, *
Hodgkin’s lymphoma	M	141			0.26, 0.003			Fat00: 0.44, *	0.41, 49, *
	F	132			0.33, *			Fat00: 0.36, *	0.39, 43, *
Kidney	M	153	0.32, *		0.54, *			Cer93: −0.26, *	0.70, 122, *
		152	0.25, *	1993; 0.39, *	0.30, *				0.70, 119, *
		154	0.37, *	1993; 0.53, *					0.67, 153, *
		153	0.36, *		0.51, *				0.64, 135, *
	F	153	0.24, *	1993; 0,21, 0.018	0.45, *			Cer93: −0.15, 0.007	0.69, 84, *
		153	0.30, *		0.57, *			Cer93: −0.21, *	0.68, 106, *
		153	0.24, *	1993; 0.34, *	0.35, *				0.67, 105, *
		153	0.37, *		0.53, *				0.64, 133, *
		154	0.28, *	1993; 0.57, *					0.62, 123, *
Laryngeal	M	157	0.68, *						0.46, 131, *
	F	139	0.31, *						0.09, 15, *
Leukemia	M	147	0.32, *	2000; 0.38, *				LE: 0.20, 0.016	0.62, 78, *
	F	151	0.37, *					LE: 0.45, *	0.52, 81, *
		146	0.32, *	2000; 0.15, 0.15				LE: 0.36, *	0.52, 52, *
Lip, oral	M	157	0.45, *		−0.20, 0.06				0.11, 10, *
	F	156	0.29, 0.002		−0.29, 0.003				0.06, 6, 0.003
Liver	M	136		1980; −0.37, *					0.13, 21, *
	F	136		1980; −0.44, *					0.19, 33, *
Lung	M	68	CigM; 0.62, *		0.33, *				0.68, 72, *
		68	CigM; 0.63, *					AF00: 0.34, *	0.67, 68, *
		68	CigM; 0.60, *		0.23, 0.04			AF00: 0.14, 0.22	0.68, 49, *
	F	71	CigF; 0.69, *					AF93; 0.26, 0.001	0.78, 123, *
		71	CigF; 0.74, *		0.21, 0.004				0.77, 118, *
Multiple myeloma	M	112		1985; 0.61, *		0.22, 0.05			0.64, 100, *
		122		1985; 0.66, *				LE: 0.17, 0.05	0.61, 97, *
	F	110		1990; 0.33, 0.003		0.49, *			0.61, 88, *
		129				0.55, *		LE: 0.28, 0.001	0.60, 85, *
Non-Hodgkin’s lymphoma	M	142				0.68, *			0.46, 120, *
	F	141				0.71, *			0.50, 140, *
Ovarian	F	138	0.25, 0.002	1990; 0.55, *					0.55, 85, *
								Sweet90:	
Pancreatic	M	132	0.42, *	1985; 0.28, 0.001			Alc93: 0.18, 0.005	0.15, 0.013	0.74, 93, *
		132	0.45, *	1990; 0.39, *				0.15, 0.013	0.73, 116, *
	F	132	0.26, 0.001	1990; 0.46, *				0.20, 0.003	0.64, 80, *
Prostate	M	137		1980; 0.52, *			Cer93: −0.23, *	Sweet90: 0.18, 0.019	0.56, 58, *
		132	0.45, *	1990; 0.39, *				0.15, 0.013	0.73, 116, *
	F	132	0.26, 0.001	1990; 0.46, *				0.20, 0.003	0.64, 80, *
Testicular	M	123		1985; 0.85, *					0.72, 312, *
Thyroid	M	146		1993; 0.78, *	−0.26, 0.009				0.36, 42, *
	F	152		1993; 0.60, *	−0.27, 0.014				0.18, 18, *

M: male; F: female; *: *p* < 0.001; sweet90: sweetener supply, 1990; sweet93: sweetener supply, 1993.

**Table 4:** Numbers of cancer types for which significant direct and inverse correlations were found.

Factor	All Countries M	All Countries F	All Countries M/F	HQ Countries M	HQ Countries F	HQ Countries M/F
Direct correlation						
Alcohol	1	1	1	1	1	1
Animal fat	2	1	2	2	1	2
Animal product energy	9	7	12	5	6	10
Fat	1	1	1	1	1	1
GDP	4	4	5	5	3	7
Latitude	6	4	6	5	6	6
Life expectancy	1	2	3	2	3	3
Lung cancer	8	11	12	8	9	10
Sweeteners	1	2	3	0	0	0
Inverse correlation						
Animal product energy	0	0	0	2	2	2
Cereals	2	1	2	2	1	2
GDP	0	1	1	0	1	1
Latitude	2	3	3	0	2	2

M: male; F: female; HQ: high quality countries.

Meat consumption has been recognized as a risk factor for cancer since at least the early 20th century. A study of cancer rates in different ethnic groups in Chicago from 1900 to 1907 found that “heavy meat eaters—Germans, Irish, Scandinavians—apparently had high rates of cancer mortality, but pasta-consuming Italians and rice-eating Chinese had low rates. Though the survey was crude, it was more thorough than many of its kind (it studied some 4600 cases over a seven-year stretch from 1900 to 1907). Its conclusions concerning the potential risk of eating too much meat crudely anticipated those of many experts eighty years later” [36,37].

The present study as well as other ecological studies found dietary animal products associated with increased risk of chronic diseases. McCarty noted that several cancers increased in frequency in Asia, in southern Europe, and among African Americans in the 20th century after the increase in dietary animal products: breast, colon, kidney ovary, pancreas, and prostate [38]. A recent ecological study of trends of Alzheimer’s disease rates in Japan correlated meat and animal products with a 15- to 25-year lag between dietary changes and prevalence of Alzheimer’s disease [39]. Iron was also suggested as a link for that finding.

Mechanisms linking animal products to risk of cancer probably include increased production of insulin, insulin-like growth factor 1, and sex hormones: “Epidemiological evidence is accumulating and suggests that the risk of cancers of the colon, pancreas, endometrium, breast and prostate are related to circulating levels of insulin, (insulin-like growth factor 1), or both” [40]. Also, iron in meat may be a risk factor for cancer through increased production of free radicals and increased oxidative stress and DNA damage [41].

The correlation of energy from animal products with risk for a number of common cancers observed in this study may meet with some skepticism, inasmuch as, in prospective or case-control studies conducted in Western countries, such a correlation is rarely reported. Correlations with red meat or flesh foods cooked in high heat occasionally emerge (possibly reflecting an impact of heme iron, mutagens, or saturated fat), but animal products *per se* are rarely incriminated in such studies. A possible explanation may be that a moderate degree of essential amino acid restriction is required for down-regulation of IGF-I, mTORC1 activity, or other yet-unknown factors which act as cancer promoters [42,43]. Such restriction may be achievable with a vegan diet of moderate protein content, but diets that incorporate meaningful quantities of high-quality animal protein would presumably be too high in essential amino acids to down-regulate cancer promotional activities. And even vegans in Western countries often have an ample intake of isolated soy protein, which is of intermediate quality and capable of boosting IGF-I levels [44,45]. So only a tiny fraction of people in Western societies are likely to achieve a degree of essential amino acid restriction capable of modulating cancer risk—and most of these are only vegan for a portion of their life span. Varying protein intake within the range typical of Western societies may have little impact on cancer promotional activities.

### 4.1. Latitude

Several previous multicountry ecological studies interpreted findings of an index for annual solar UVB dose, which is strongly inversely correlated with latitude, as an index of vitamin D production. Such studies found inverse relations between that index and incidence rates for a number of types of cancer. Those cancers included bladder [46], brain [47], breast [48], colorectal [49], endometrial [50], kidney [51], lung [52], ovarian [53], pancreatic cancer [54], and leukemia [55]. However, under the Bonferroni principle (*p* must be < 0.05/*n*, where *n* is the number of factors for significance to be claimed), the UVB index was significantly correlated with only bladder, brain, kidney, and lung cancer. This study confirmed these findings.

As to why this study did not find more cancers to have significant increases with increasing latitude, it appears that if animal products are significantly correlated with cancer incidence rates, it is less likely that latitude is. This seems to be the case for breast, corpus uteri, ovarian, and pancreatic cancer. For kidney cancer, when energy from animal products is included, the correlation for latitude is lower than when cereals are included.

In single-country studies, latitude is often a good index of solar UVB doses [56,57,58]. However, in the United States, considerable asymmetry exists in solar UVB doses owing to the geography [59]. However, the interpretation of latitude as an index of vitamin D production globally is questionable. The primary reason is that skin pigmentation varies with solar UV doses where people have lived for many generations [60,61] so that serum 25-hydroxyvitamin D [25(OH)D] levels do not vary much globally. Three reviews found that country mean serum 25(OH)D levels are near 50 nmol/L in nearly every country [62,63,64]. The nearly constant serum 25(OH)D with respect to latitude is reasonable since if large differences existed, risk of vitamin D—sensitive diseases would vary considerably with respect to latitude. A pronounced increase of multiple sclerosis prevalence occurs with increasing latitude [65]. One study inversely correlated both solar UV dose and vitamin D with risk of multiple sclerosis [66].

The inverse correlation for cervical cancer can be understood in terms of higher risk of infections at low latitudes, and that for lip cancer in that UVB is a risk factor for lip cancer [22].

An alternative interpretation of latitude is that it may be an index of various modeled and/or unmodeled factors such as infection, living in urban regions, and eating processed food. For example, *Toxoplasma gondii* infection rates increase with latitude, and *T. gondii* is a risk factor for brain cancer [67].

### 4.2. GDP

Studies have often correlated per capita GDP with increased risk of chronic diseases, such as cancer [6] and diabetes [68]. Several possible reasons may account for why cancer rates are higher in countries with higher GDP: more time spent indoors, less time engaged in physical exercise [69], greater consumption of processed foods [70], greater life expectancy [71], more chemical pollution exposure, and reduced risk of infectious diseases [72,73].

### 4.3. Life Expectancy

**Figure 1:**
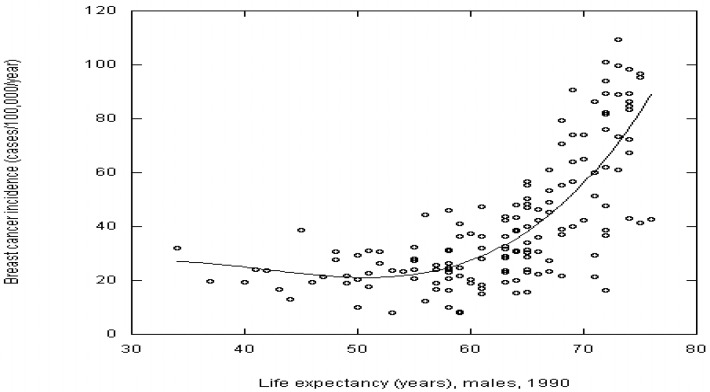
Breast cancer incidence rates for 2008 *vs*. life expectancy for males in 1990 with a third order fit.

Cancer risk does increase with life expectancy for most cancers in this study, such as breast and colorectal cancer (see Figure 1). However, life expectancy is significantly correlated with only three cancers: brain cancer, leukemia, and multiple myeloma. Why life expectancy is not significantly correlated with other cancers in this study is unclear.

### 4.4. Individual Cancers

Table 5 lists journal papers supporting the findings in Table 2 and Table 3. Findings from this ecological study generally agree with the journal literature for most factors for most cancers studied. Some results in the journal literature were not supported in this study, such as alcohol for several cancers, animal products for colorectal cancer, and latitude for many cancers. These failures indicate that such ecological studies can identify only up to three or four cancer risk-modifying factors as having significant correlations with cancer incidence rates in the best model for the particular cancer. Thus, findings by such ecological studies do not offer evidence that nonsignificant risk-modifying factors are not related to cancer risk, just that they seem less important than other factors.

This study found no significant results for esophageal, gallbladder, and stomach cancer. Esophageal and stomach cancer have infections as important risk factors (see Table 5).

Studies have inversely correlated white meat [74] and fish [75] with risk of liver cancer, whereas other studies directly correlated liver cancer risk with intake of red and processed meat [74,75].

The directly correlation of latitude with melanoma risk is related to skin pigmentation. Melanoma incidence rates for people living in their ancestral homelands below 35° latitude are very low (mean value, ~2 cases/100,000/year) but rise rapidly at higher latitudes (regression mean, ~14 for >60° latitude). The main reason for this latitudinal variation is variation in skin pigmentation, which has adjusted for protection against adverse effects of solar UV while permitting adequate production of vitamin D [56]. Australia and New Zealand have the highest melanoma incidence rates and are populated primarily by people who can trace their ancestry to northern Europe. GDP’s direct correlation with melanoma incidence rates may be related to increased travel to sunny locations for those living in the wealthiest countries. Studies in Norway and the United States have correlated air travel with risk of melanoma [76,77].

**Table 5:** Journal literature supporting findings in this study.

Cancer	Animal Products	Smoking	GDP per Capita	Latitude *	Alcohol	Misccellaneous	Infection
Bladder	[78,79]	[80,81]	[82]	[46] UVB			
Brain	[83]	[84]		[47,67]			[67,85]
Breast	[86,87]		[82,88]	UVB			
Cervix		[80]					[89]
Colorectal	[78,90,91]	[80]	[49,82,88]	UVB	[92,93]	Sugar [94]	[89]
Corpus uteri	[78]	[95]		uvb		Sugar [96]	
Esophageal	[90,91,97]	[80,81]		uvb	[92]		[98]
Gallbladder				uvb			
Gastric		[81]		uvb			[99]
Hodgkin’s lymphoma	[100]			uvb			[101]
Kidney	[79]	[102]	[82]	[51,103] UVB		Cereals [104,105]	
Laryngeal		[80,81]		uvb	[92]		[106]
Leukemia	[107]	[107]		[55]			
Lip		[108]		[108] (direct)			
Liver	[74,90]	[81,102]					[109]
Lung	[87,91,110]	[81]	[88]	[52] uvb			
Mult. Myel.	[100,111]						[112]
Non-Hodgkin’s lymphoma	[113,114]		[82,88]	UVB			[115]
Ovarian	[78,116,117]	[118]	[82]	[53] UVB			
Pancreatic	[78,87,119]	[80,81]	[88]	[54] UVB	[92,120]	Sugar [94]	
Prostate	[79]	[121] (inverse)	[82,88]	uvb	[122]	Sugar [94], rice [121]	
Testicular	[79]					Fat [123]	
Thyroid	[124]			uvb			

UVB: strong support for UVB from single-country studies [9,56,57,58,125]; uvb: moderate support.

### 4.5. Strengths and Limitations of This Study

Ecological studies such as those reported here have several strengths and limitations. Strengths include that such ecological studies involve many cases, permitting investigation of many cancers and consideration of many factors. Also, since data exist for several indices of cancer risk-modifying factors over many years, ecological studies can investigate the effect of delay between risk-modifying factor and cancer incidence.

Limitations include that the data are for country populations as a whole, so values for risk-modifying factors may not accurately reflect risk factors on individuals who develop cancer. For example, solar UVB doses vary widely in mid-latitude countries with large latitude ranges. Also, dietary intake varies widely by individual. Further, dietary supply data are based on food that enters the consumer supply and does not account for waste, loss, and spoilage. This study assumes that consumption rates are a similar fraction of consumer supply for each country, probably around 70%. Also, country-averaged data for some cancer risk-modifying factors, such as rates for obesity, physical exercise, and diseases that may compete with risk of cancer (e.g., cardiovascular disease and infectious diseases), are either not available or were not included in the analysis. In addition, there are cancer risk-modifying factors that are particular to a few countries or regions within countries. Examples of such factors include poor sewage disposal, which is a risk for *Helicobacter pylori* infection and gastric cancer [126], arsenic in drinking water and risk of renal cancer [127], industrial pollution, a risk factor for several types of cancer [128], betel quid chewing and risk of head and neck cancers [129].

## 5. Conclusions

This multicountry ecological study of cancer incidence rates in 2008 found that lung cancer incidence rates, supply of energy derived from animal products, latitude (an index for solar UVB doses), and per capita GDP had the largest number of significant correlations with various cancers. Alcoholic beverages, animal fat, life expectancy, and sweeteners had significant correlations with a few cancers. This study found inverse correlations for cereals, per capita GDP, and latitude for one to three cancers each. For all countries, the models explained 9%–84% of the variance, with median values of 62% for males and 54.5% for females. For countries with high-quality data, the models explained 6%–75% of the variance, with median values of 55% for males and 41.5% for females. Since most factors considered are environmental, rather than genetic, one may reasonably conclude that environmental causes of cancer contribute much more to cancer risk than do genetic factors, and thus lifestyle changes could reduce much of the cancer burden.

## Appendix

**The 87 countries with** **level 1 or 2 quality and life expectancy for males in 1990 >60 years:** Albania, Argentina, Armenia, Australia, Austria, Azerbaijan, Bahamas, Barbados, Belarus, Belgium, Belize, Brazil, Brunei, Bulgaria, Canada, Columbia, Costa Rica, Croatia, Cuba, Cyprus, Czech Republic, Denmark, Dominican Republic, Ecuador, Egypt, El Salvador, Estonia, Fiji, Finland, France, Georgia, Germany, Greece, Guatemala, Guyana, Hungary, Iceland, Iran, Ireland, Israel, Italy, Jamaica, Japan, Kazakhstan, Korea (Republic), Kuwait, Kyrgyzstan, Latvia, Lithuania, Luxembourg, Macedonia, Malaysia, Mauritius, Mexico, Mongolia, Netherlands, New Zealand, Nicaragua, Norway, Panama, Paraguay, Peru, Philippines, Poland, Portugal, Romania, Russia, Slovakia, Slovenia, South Africa, Spain, Sri Lanka, Suriname, Swaziland, Sweden, Switzerland, Syria, Tajikistan, Trinidad and Tobago, Turkey, Turkmenistan, Ukraine, United Kingdom, United States, Uruguay, Uzbekistan, Venezuela.

**The other 70 countries:** Algeria, Angola, Bangladesh, Benin, Bolivia, Botswana, Burkina Faso, Burundi, Cambodia, Cameroon, Cape Verde, Central African Republic, Chad, Chile, China, Comoros, Djibouti, Ethiopia, French Polynesia, Gabon, Gambia, Ghana, Guadeloupe, Guinea, Guinea-Bissau, Honduras, India, Indonesia, Iraq, Ivory Coast, Jordan, Kenya, Laos, Lebanon, Lesotho, Libya, Madagascar, Maldives, Mali, Malta, Martinique, Mauritania, Moldova, Morocco, Mozambique, Myanmar, Namibia, Nepal, New Caledonia, Niger, Nigeria, Pakistan, Papua New Guinea, Reunion, Saudi Arabia, Senegal, Sierra Leon, Solomon Islands, Sudan, Tanzania, Thailand, Togo, Tunisia, Uganda, United Arab Republic, Vanuatu, Viet Nam, Yemen, Zambia, Zimbabwe.
